# Mediating effects of shoulder-arm exercise on the postoperative severity of symptoms and quality of life of women with breast cancer

**DOI:** 10.1186/s12905-020-00968-w

**Published:** 2020-05-11

**Authors:** I-Hui Chen, Chia-Hui Wang, Shu-Yi Wang, Sue-Yueh Cheng, Tzu-Jou Yu, Shu-Fen Kuo

**Affiliations:** 1 School of Nursing, College of Nursing, Taipei Medical University, 250 Wuxing Street, Taipei, 11031 Taiwan; 2 Loretto Heights School of Nursing, Rueckert-Hartman College for Health Professions, Regis University, 3333 Regis Boulevard, G-8, Denver, CO 80221-1099 USA

**Keywords:** Breast cancer, Shoulder-arm exercise, Severity of symptoms, Quality of life (QoL)

## Abstract

**Background:**

The postoperative severity of symptoms among women with breast cancer affects their quality of life (QoL). Although it is recommended that performing shoulder-arm exercise 30 min/day can alleviate symptoms and improve the QoL, there is little research on the mediating effects of performing shoulder-arm exercise 30 min/day on the postoperative severity of symptoms and QoL among patients with breast cancer.

**Methods:**

A cross-sectional study was conducted 2 ~ 4 months after surgery on women diagnosed with breast cancer but with no distant metastasis and who had undergone breast cancer surgery for the first time. A structured questionnaire was employed which included a severity of symptoms scale, performing shoulder-arm exercise for 30 min/day, a QoL scale, demographic characteristics, and medical status.

**Results:**

In total, 117 women with breast cancer completed the survey. The severity of symptoms and performing shoulder-arm exercise 30 min/day separately affected the QoL (B = -0.447, standard error (SE) = 0.050, *p* < 0.001; B = 15.666, SE = 4.542, *p* = 0.001, respectively). In model 3, performing shoulder-arm exercise for 30 min/day played a partial mediating role in the relationship of the severity of symptoms and QoL (*R*^2^ = 0.51, *F* = 5.41, *p* < 0.001).

**Conclusions:**

During 2 ~ 4 months after surgery, regular shoulder-arm exercise for 30 min/day could decrease the effect of the severity of symptoms on the QoL among women with breast cancer. Clinical healthcare providers may inform and educate patients as to the benefits of regular shoulder-arm exercise for 30 min/day.

## Background

Breast cancer is the most frequently diagnosed cancer and also the leading cause of cancer deaths among women worldwide [1]. In order to prolong survival times, women with breast cancer usually undergo surgery (i.e., a modified radical mastectomy (MRM) or breast-conserving therapy (BCT)) [2]. Much research investigating symptom interference has been conducted in breast cancer patients, especially on the physical well-being and quality of life (QoL) within 15 months after a diagnosis [3]. Commonly reported severe symptoms that interfere with patients’ lives include fatigue, pain, sleep disturbances, lymphedema, and arm weakness after surgery to 3 months of follow-up because of chronic and progressive swelling and recurrent skin infections. A past study showed that the worst QoL was observed in the first month after surgery, and having less-severe symptoms was one of the predictors of positive QoL trends in 3 months following surgery [4]. When assessing the severity of symptoms, it is important to gauge to what extent symptoms interfere with a patient’s life and QoL in order to understand how patients manage and cope with their symptoms.

The ability to perform activities of daily life (ADLs) and levels of functionality are essential to determining the QoL of breast cancer survivors. In particular, adverse effects of treatment (e.g., pain and fatigue) can interfere with one’s functional capacity (FC) and directly affect one’s QoL [5]. So, persistent FC and QoL should be discussed longitudinally among breast cancer patients after surgery, especially including all activities and exercises [6].

Recent comprehensive research indicated that shoulder-arm exercise training after surgical treatment may prevent and reduce complaints of lymphedema and limited shoulder joint movement, thereby simultaneously promoting the QoL [7–12]. A past study showed that there was higher severity of symptoms at 3 months after surgery which was negatively related to shoulder-arm exercise and shoulder function [13]. But the rate of performing shoulder-arm exercise in patients after breast cancer surgery was only 37.1% [14]. Reasons for developing lymphedema caused by a lack of shoulder-arm exercise were a failure to follow recommendations and insufficient information [15]. Exercise rehabilitation can be an effective self-management strategy to control cancer treatment-related symptoms and promote the QoL, including shoulder range of motion (ROM) [6, 7]. The benefits of shoulder-arm exercise are imperative to ameliorate the effects of surgery, chemotherapy, and radiotherapy in breast cancer patients. However, no study has been conducted to investigate the potential mediating effect of shoulder-arm exercise on the severity of symptoms and QoL. We attempted to extend our knowledge of symptom experiences of patients with breast cancer in order to improve their QoL in the postoperative 2 ~ 4-month period. To understand this phenomenon, the aims of this study were to investigate relationships of performing shoulder-arm exercise with the postoperative severity of symptoms and QoL among women with breast cancer during 2 ~ 4 months after surgery.

## Methods

### Study design and procedures

This study used a cross-sectional approach. Research participants were from the outpatient departments of two teaching hospitals, and procedures were reviewed and approved by the two hospitals’ Institutional Review Boards (Cathay General Hospital-IRB, and Taipei Medical University Hospital-IRB). All participants were provided an explanation of the study, and written informed consent was obtained prior to recruitment. The questionnaire was filled out and collected in a clinical setting.

### Participants and sample size

Inclusion criteria for the study were women: (1) with breast cancer who had received sentinel node biopsies and a pathological report indicating no distant metastasis and who had received axillary node dissection; (2) who had not received breast reconstruction in those with shoulder-arm restriction within 6 weeks of having received surgery; (3) who were currently in the 2nd ~ 4th months after cancer surgery; (4) who were ≥ 18 years of age and with clear consciousness; and (5) who were able to communicate in Mandarin or Taiwanese. Exclusion criteria were women who had a history of mental illness.

To obtain a power of 0.80, with an effect size, F^2^, of 0.15, alpha of 0.05, and number of predictors of 9, the sample size calculation for statistical significance with a linear multiple regression was performed. The total sample size needed was determined to be 114. All participants received postoperative education on performing shoulder-arm exercises, and began performing shoulder-arm exercises 30 min/day, including climbing a wall, rope movement, pulley movement, and shoulder-angle movement [7]. The study initially approached 123 postoperative women with breast cancer who met the inclusion and exclusion criteria. Six of the women expressed no interest in participating, and ultimately 117 women with breast cancer completed the questionnaire. The attrition rate was 91%.

### Demographic characteristics and medical status

Demographic data included age and educational level. The medical status included tumor size, lymph node involvement, type of surgery (MRM or BCT), time after surgery, and treatment received after surgery.

### Functional living index-Cancer (FLIC)

The FLIC is a tool [4] for assessing a patient’s QoL which emphasizes the extent cancer and its related treatments affect patients’ normal functions in all areas of life. There are 22 questions in five domains, including physical functioning, mental functioning, social functioning, general health/well-being, and gastrointestinal symptoms, which use a 7-point Likert scale for scoring; the total score ranges 0 ~ 154. The Chinese version of the FLIC had strong psychometrics for various cancer patients, including breast cancer patients [14, 16]. Cronbach’s α in this study was 0.93, thus showing good reliability of the FLIC.

### Symptom severity scale (SSS)

The SSS (26 questions) was used to assess the severity of symptoms experienced by breast cancer patients [17]; it utilizes a 0 ~ 10 visual analog scale (VAS), with 0 being the complete absence of a symptom and 10 representing the worst possible effect a patient could imagine [17]. A higher score indicates more-severe symptoms. The scale contains self-reported assessments of 26 symptoms following cancer treatments after surgery, such as nausea, vomiting, lack of appetite, pain, insomnia, tiredness, constipation, difficulty urinating, difficulty breathing, coughing, bloating, dry mouth, mouth ulcers, restlessness, inability to concentrate, appearance changes, bleeding, tremors, fever, numbness, chest tightness, stomach burning, arm swelling, hot flushes, sexual problems, and restricted arm movement after breast cancer treatment. The SSS had strong psychometrics for breast cancer patients [17]. Cronbach’s α coefficient in this study was 0.92.

### Performing the shoulder-arm exercise variable

Performing the shoulder-arm exercise for 30 min/day was assessed by one item and modified from the item developed by Cheng and her associates [13]. The shoulder-arm exercises focus on the levator scapulae, upper trapezius, pectoralis major, and medial and lateral rotator muscles of the shoulder [10, 18], and include climbing a wall, rope movement, pulley movement, and shoulder-angle movement each day for 30 min [7, 19] Breast cancer patients received shoulder-arm exercise education after surgery in the hospital from a primary nurse and provided regular feedback on the frequency of performing shoulder-arm exercise to doctors in outpatient clinics. If participants performed shoulder-arm exercise 30 min every day, they answered “Yes”; otherwise, they answered “No” [13].

### Statistical analysis

A descriptive analysis was performed on demographic characteristics, the medical status, and main factors (severity of symptoms, performing shoulder-arm exercise 30 min/day, and QoL,) An analysis of variance (ANOVA), Pearson’s correlation, and *t*-test were used to examine differences in relationships among demographic characteristics, medical status, and QoL. Performing shoulder-arm exercises for 30 min/day was examined as a mediator of the relationship between symptom severity and QoL, using a multiple regression analysis [20]. The significant confounding factor was first put into the regression model to control the effect on QoL, which was treatment received after surgery. In the next step, a mediation analysis was performed to investigate if performing shoulder-arm exercises for 30 min/day mediated the effect of symptom severity on the QoL. Path A was to determine if the severity of symptoms (independent variable) had a significant effect on performing shoulder-arm exercises for 30 min/day (the mediation variable). Because the mediator was a categorical variable, a logistic regression was used for path A [21]. Path B was to determine if performing shoulder-arm exercises for 30 min/day (mediation variable) had a significant effect on the QoL. Path C was to determine if the severity of symptoms (independent variable) had a significant influence on the QoL. Path C′ was to determine whether a change in the severity of symptoms related to QoL was indirectly affected by performing shoulder-arm exercises for 30 min/day (mediation variable). If the severity of symptoms and performing shoulder-arm exercises for 30 min/day were two significant predictors of the QoL, performing shoulder-arm exercises for 30 min/day would be a partial mediating variable. In addition, if performing shoulder-arm exercises for 30 min/day was the only predictor of the QoL, it would be a mediating variable [20]. All statistical analyses were conducted using SPSS (vers. 19.0 for Windows). A *p* value of < 0.05 indicated that the findings were significant.

## Results

### Characteristics of participants

Results of the descriptive analysis of women with breast cancer are shown in Table 1. The majority of women (40.2%) were 50 ~ 59 years old, had a senior high school education (35%), had tumor size at the T1 stage (43.6%), and had no lymph node involvement (58.1%). Eighty women (68.4%) had undergone MRM surgery, and 37 (31.6%) had undergone BCT. Most of the participants who were receiving treatment after surgery were undergoing chemotherapy (77.8%). The severity of symptoms and QoL scores were 40.52 (SD = 33.7, range 0 ~ 154) and 107.35 (SD = 23.12, range 44 ~ 153), respectively (Table 1).

**Table 1:** Descriptive analysis of participants (*N* = 117)

Variable	*n* (%) /Mean (SD)	Range
Age (years)		
30 ~ 39	15 (12.8)	
40 ~ 49	40 (34.2)	
50 ~ 59	47 (40.2)	
60 ~ 69	15 (12.8)	
Educational level		
Elementary school	12 (10.3)	
Junior high school	16 (13.7)	
Senior high school	41 (35.0)	
University	31 (26.5)	
Graduate school	17 (14.5)	
Tumor size		
T1 (≤2 cm)	51 (43.6)	
T2 (> 2 cm and < 5 cm)	44 (37.6)	
T3 (≥5 cm)	22 (18.8)	
T4 the extent to which it has grown into neighboring breast tissue	0 (0)	
Lymph node involvement		
0 = No	68 (58.1)	
1 = Yes	49 (41.9)	
Surgery		
MRM	80 (68.4)	
BCT	37 (31.6)	
Time after surgery (days)		
30 ~ 60	43 (36.8)	
61 ~ 90	48 (41.0)	
91 ~ 107	26 (22.2)	
Treatment received after surgery		
0 = none	12 (10.3)	
1 = Chemotherapy	91 (77.8)	
2 = Radiotherapy	4 (3.4)	
3 = Hormone treatment	10 (8.5)	
Severity of symptoms	40.52 (33.7)	0 ~ 154
Performing shoulder-arm exercises 30 min/day		
0 = No	84 (71.8)	
1 = Yes	33 (28.2)	
Quality of life	107.35 (23.12)	44 ~ 153

*SD* Standard deviation, *MRM* Modified radical mastectomy, *BCT* Breast-conserving therapy

In addition, the treatment received after surgery was a predictor of the QoL (*F* = 5.410, *p* = 0.002). Women who had received chemotherapy had a significantly lower QoL than women who had received no chemotherapy in this study (*t* = 3.467, *p* = 0.001). Other characteristics and medical status factors were not predictors of the QoL, including age, educational level, tumor size, lymph node involvement, type of surgery, or time after surgery.

### Mediation analysis

**Fig. 1:**
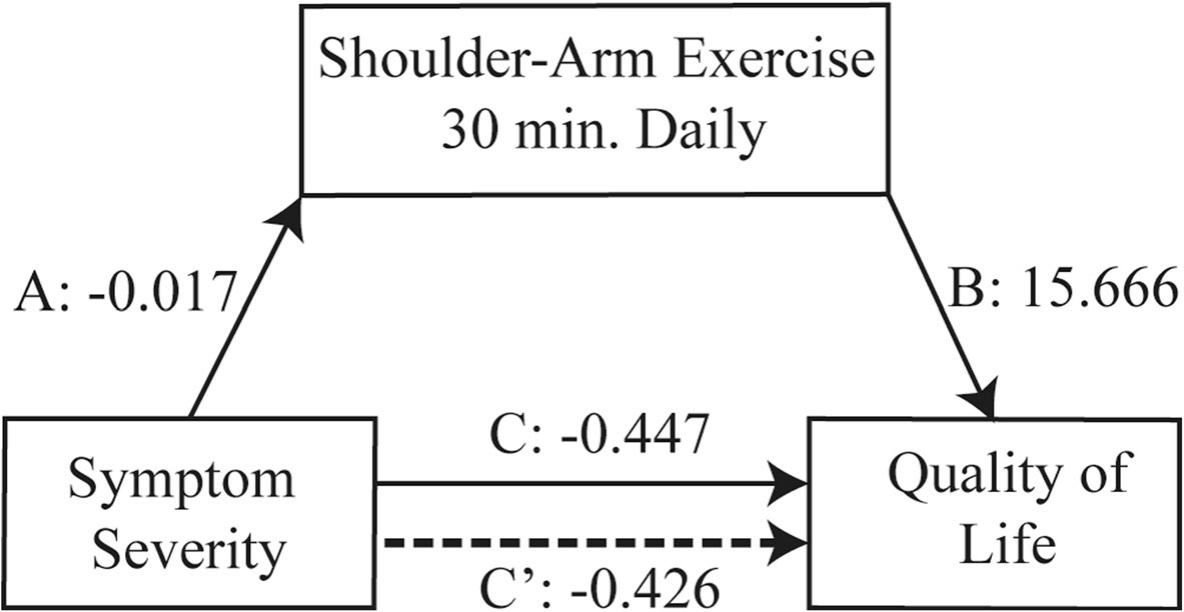
Mediating effect of shoulder-arm exercise on the severity of symptoms and quality of life. Non-standardized path coefficients are presented. A partial mediating effect was found for performing shoulder-arm exercises for 30 min/day

Figure 1 shows that the severity of symptoms was a significant predictor of performing shoulder-arm exercises for 30 min/day (B = -0.017, SE = 0.008, *p* = 0.023) on path A, and performing shoulder-arm exercises for 30 min/day was a significant predictor of the QoL (B = 15.666, SE = 4.542, *p* = 0.001) on path B. Table 2 presents unstandardized coefficients from the regression model that predicted the QoL from the treatment received after surgery, severity of symptoms, and performing shoulder-arm exercises for 30 min/day. Results of model 1 indicated that when receiving chemotherapy after surgery, women had a significantly lower QoL compared to women who had no treatment after surgery (B = -22.659, SE = 6.728, *p* = 0.001). Model 2 indicates that the severity of symptoms predicted the QoL (B = -0.447, SE = 0.050, *p* < 0.001). When performing shoulder-arm exercises for 30 min/day was entered into model 3, the result of the beta weight of severity of symptoms was still significant (B = -0.426, SE = 0.050, *p* < 0.001), and shoulder-arm exercises remained a significant predictor of the QoL (B = 8.255, SE = 3.483, *p* = 0.02). So performing shoulder-arm exercises for 30 min/day partially mediated the effect of the severity of symptoms on the QoL among breast cancer patients during 2 ~ 4 months postoperatively (Table 2).

**Table 2:** Mediating effect of performing shoulder-arm exercises for 30 min/day on the relationship between severity of symptoms and quality of life (*N* = 117)

Effect	1	2	3
(Constant)	126.000 (6.331)***	133.171 (4.957)***	129.417 (5.117)***
Treatment received after surgery (reference = none)			
1 = Chemotherapy	−22.681 (6.736)**	−9.578 (5.411)	−8.846 (5.315)
2 = Radiotherapy	−19.750 (12.663)	3.842 (10.140)	4.107 (9.943)
3 = Hormone treatment	−4.000 (9.391)	−5.196 (7.254)	−5.004 (7.113)
**Independent variable**			
Severity of symptoms		−0.446 (0.051)***	−0.425 (0.050)***
**Mediating variable**			
Performing shoulder-arm exercises 30 min/day (reference = No)			8.204 (3.499)*
Adj. *R*^2^	0.10	0.46	0.48
F (*p*)	5.408 (0.002)	26.165 (< 0.001)	22.873 (< 0.001)

Standard errors are in parentheses

* *p* < 0.05; ** *p* < 0.01; *** *p* < 0.001

## Discussion

The main purpose of this study was to investigate whether regularly performing shoulder-arm.

exercises for 30 min/day mediated effects of the severity of symptoms on the QoL after surgery in women with breast cancer. In a majority of trials, QoL is only included as a secondary endpoint [22]. In reality, QoL is one of only two clinically relevant patient endpoints [23]. As such, the QoL endpoint should always be a primary endpoint in any clinical trial. More importantly, any trial that evaluates changes in practices should be based on QoL endpoints, and not surrogate endpoints, like the most commonly used progression-free survival (PFS).

Study results showed that the severity of symptoms was a predictor of the QoL during the early period after surgery, which corresponds to past literature that included physical and psychological symptoms of combining surgery and other treatments [14]. In particular, elevated symptom scores were most commonly reported within 6 months after a diagnosis [17]. In addition, the severity of symptoms was a predictor of performing shoulder-arm exercises for 30 min/day in this study. When shoulder-arm exercise was added to the regression model of the severity of symptoms on the QoL, the beta weight of the severity of symptoms slightly decreased and had a significant effect on the QoL. So, shoulder-arm exercises were a partial mediator between the relationship of severity of symptoms and the QoL. This demonstrated that shoulder-arm exercises produced an effect on the severity of symptoms on the QoL, so it is important to encourage and support regular shoulder-arm exercises in the early period after breast surgery [7].

The effects of arm disability on the QoL had the same meanings as shoulder-arm exercise on the QoL in this study. Because regular shoulder-arm exercises can prevent lymphedema, the QoL among women who underwent breast surgery could be maintained. QoL is a good indicator of breast cancer women’s mortality, because most cancer survivors experience declines in physical activity and QoL in response to poor treatment compliance [7]. Despite past studies examining exercise-induced functional improvements of the shoulder during 14 days after breast surgery [7, 8], regular shoulder-arm exercises were first demonstrated in this study to reduce the effect of the severity of symptoms on the QoL. The American Cancer Society recommends that it is important to do shoulder-arm exercises after surgery to strengthen the arm and shoulder and prevent breast cancer-related lymphedema after surgery [24, 25]. Based on these study results, protective exercise education about preventing lymphedema should be taken seriously and provided separately from other regular postoperative education.

The concept of a healthy life should be emphasized after breast-surgery treatment [8]. Shoulder-arm exercises are one set of exercises recommended for breast surgery patients and can persist for a long time before other exercise recommendations; the timing of rehabilitation exercise to prevent lymphedema after surgery depends on limitations of the shoulder and arm [6, 8, 17]. Even if cancer treatment-related symptoms of breast cancer patients are reduced by supplementing home exercises after an oncologist’s recommendation (for example fatigue or shoulder ROM) [7, 26], all of the women stopped doing arm exercises when their arms felt better before lymphedema development due to insufficient information and failure to follow recommendations [15]. Outcomes of exercise-related compliance behaviors on the severity of symptoms and the QoL have not been well studied. So details of the exercise process (e.g., the timing of exercise, frequency, etc. and how to increase ROM) should be explored and designed into tailored educational interventions based on symptom severity and QoL from the early post-surgical period to the entire life of breast cancer patients. In addition, interpersonal support and situational support (e.g., a sharing group) can enhance a person’s willingness to perform healthy behaviors, even if they have severe symptoms and lack the time [14, 27]. Although the shoulder-arm exercise is routine education in clinical settings after breast cancer surgery, clinical staff should pay more attention to the completion rate of performing shoulder-arm exercises, and inform patients as to the importance of performing shoulder-arm exercises for 30 min/day and not stopping when their arms feel better early after surgery, especially when undergoing chemotherapy.

There were some limitations in this study. First, this was postoperative research of women with breast cancer from two hospitals in northern Taiwan, so results cannot be inferred to all women after breast cancer surgery. Second, this study lacked baseline data and a comparator, which severely limit interpretation of the QoL. Well-designed and well-executed clinical trials are needed to draw convincing conclusions. Third, effects of demographic and medical-treatment factors on breast-cancer treatment symptoms and the QoL should be repeated and demonstrated with a larger sample, and should include age, educational level, and breast-cancer treatments after surgery. Breast cancer-related symptom clusters may be related to stage and multiple treatments [5]. Fourth, the roles of other manual lymphatic drainage classes which focus on breast-cancer treatment symptoms and QoL should be verified. Fifth, future studies should attempt to understand relationships of other factors on symptom severity and the QoL. For example, the Karnofsky Performance Score (KPS) was significant relative to fatigue and QoL [15, 28]. And the KPS was significantly related to QoL among women without and those with locoregional and distant metastases [29]. Finally, the study’s cross-sectional design prevented us from observing cause-effect relationships among symptom severity, regular shoulder-arm exercises, and QoL. Prospective studies are needed to confirm links of symptom severity (especially lymphedema) with regular shoulder-arm exercises and the QoL (as a primary endpoint) among women after breast cancer surgery.

## Conclusions

This study demonstrated the strength of the mediating role of shoulder-arm exercises that could underlie the relationship between severity of symptoms and QoL among postoperative breast cancer women who received axillary node dissection during surgery. The current findings may be of clinical importance because they suggest the need to routinely consider postoperative shoulder-arm exercises for women with breast cancer. This study also fills in a gap in the literature related to the QoL of women with breast cancer suffering from lymphedema. Future studies can focus on prospective follow-up QoL studies, with larger sample sizes and multiple-time points. In addition, more studies (mainly clinical trials) need to include QoL as a primary endpoint, and not just as a secondary endpoint.

## Data Availability

The datasets analyzed during the current study are available from the corresponding author on reasonable request.

## Abbreviations

BCT: Breast-conserving therapy
FLIC: Functional Living Index-Cancer
KPS: Karnofsky Performance Score
MRM: Modified radical mastectomy
QoL: Quality of life
ROM: Range of motion
SSS: Symptom Severity Scale
VAS: Visual analog scale
